# Not All Heart Failure Improves With Diuresis: A Lesson in Left Ventricular Assist Device Physiology

**DOI:** 10.1016/j.case.2025.01.003

**Published:** 2025-03-06

**Authors:** Giancarlo Saldana, Steve Mazzone, Kyle Hipke, Mark N. Belkin, Karima Addetia

**Affiliations:** Heart and Vascular Center, University of Chicago, Chicago, Illinois

**Keywords:** Left ventricular assist device, Tamponade, Outflow graft, Fluid collection, Heart failure, Multimodality imaging

## Abstract

• LVADs are a key destination therapy for many patients with end-stage heart failure.
• Multimodal imaging, including TTE and CCT, is key in diagnosing LVAD complications.
• Outflow graft seromas may cause chamber compression as a rare LVAD complication.

## Graphical abstract

**Figure:**
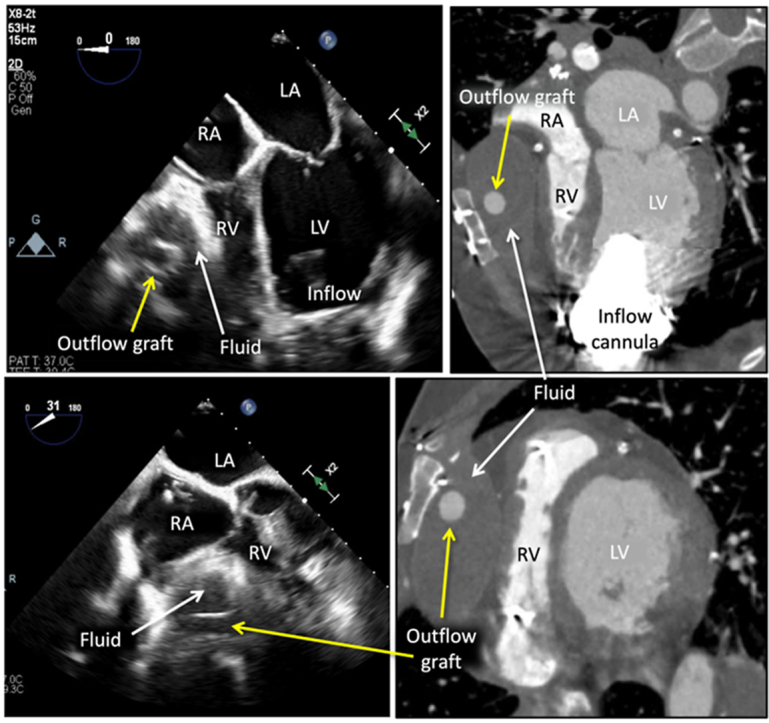

## Introduction

Although heart transplantation is considered the optimal therapy for end-stage heart failure, there are not enough donors to meet the rising demand. For patients with refractory heart failure who require additional hemodynamic support, left ventricular assist devices (LVADs) may be an option. Transthoracic echocardiography (TTE) remains the initial modality of choice for routine LVAD evaluation and speed optimization. On the basis of clinical trial data, 25% of patients with continuous-flow LVADs experience New York Heart Association class II to IV symptoms, likely due, in large part, to inadequate left ventricular unloading.^1^ We present the case of a patient who developed cardiac tamponade 4 years after LVAD implantation, in whom multimodality imaging was invaluable in determining the unusual cause and instrumental in altering the course of the patient’s care.

## Case Presentation

**Figure 1:**
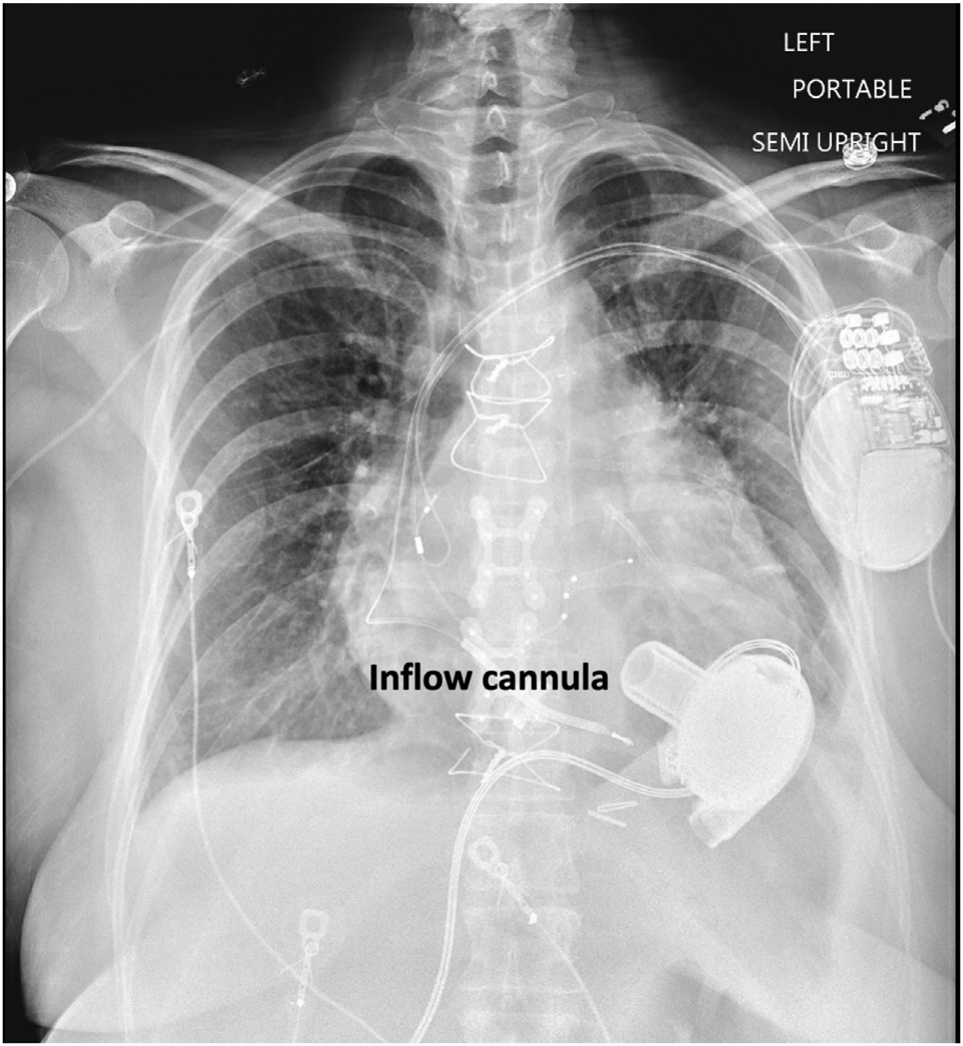
Portable, semiupright posterior-anterior chest radiograph at admission demonstrates mild, diffuse bilateral pulmonary edema and stable, unchanged position of the LVAD inflow cannula compared with prior; also shown is the left-sided cardiac resynchronization therapy device with defibrillator and right arm peripherally inserted central catheter.

A 50-year-old patient with a history of ischemic cardiomyopathy, who had undergone LVAD implantation 4 years prior as destination therapy, presented to the hospital with a 1-week history of severe dyspnea. Their post-LVAD course was complicated by chronic driveline infections, including an abscess that required surgical drainage, and they were on lifelong antibiotic therapy. A family member reported that the patient was occasionally “turning blue around the mouth.” Initial vital signs showed a mean arterial pressure of 85 mm Hg, a heart rate of 99 beats/min, and an oxygen saturation of 83% on room air. The physical examination was notable for elevated jugular venous pressure, bibasilar lung crackles, and +1 lower extremity pitting edema that were cool to the touch. Chest radiography at the time of admission demonstrated mild bibasilar opacities consistent with pulmonary edema, as well as bilateral pleural effusions (Figure 1). Interrogation of the LVAD device revealed occasional flow alarms but no power surges, and the rotor speed was recorded at 2,860 rotations/min. Laboratory data showed a pro–brain natriuretic peptide level of 7,612 pg/mL (the last recorded level was 734 pg/mL several months earlier after treatment for an acute decompensated heart failure episode), mildly elevated liver enzymes, and a normal creatinine level. Given the high concern for acute decompensated heart failure and early cardiogenic shock, the patient was admitted to the cardiac intensive care unit and treated with intravenous diuretic therapy. The following day, the patient underwent right heart catheterization (RHC) for evaluation of filling pressures and cardiac function, as well as placement of a pulmonary artery catheter. Invasive hemodynamics (Table 1) demonstrated elevated biventricular filling pressures with a low cardiac index, suggestive of cardiogenic shock. Given these findings, the patient remained in the intensive care unit and was treated with aggressive diuresis.

**Table 1:** RHC results, LVAD speed 2,860 rotations/min

Parameter	Value
Right atrial pressure, mm Hg	25
Right ventricular systolic/diastolic pressure, mm Hg	87/30
PA systolic/diastolic pressure, mm Hg	82/45
PCWP, mm Hg	36
PA saturation, %	40.9
Fick cardiac output, L/min	2.05
Fick cardiac index, L/min/m	1.4
SVR, dyne · s ·cm^−5^	2,384

*PA*, Pulmonary artery; *PCWP*, pulmonary capillary wedge pressure; *SVR*, systemic vascular resistance.

**Figure 2:**
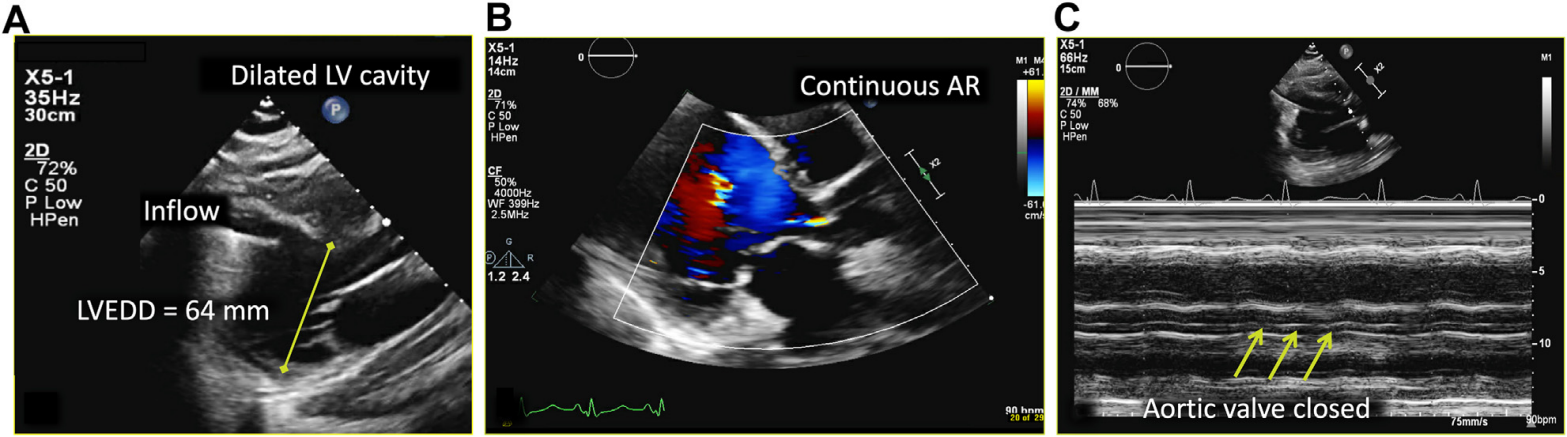
Two-dimensional TTE at admission, parasternal long-axis view without **(A)** and with **(B)** color flow Doppler and M-mode **(C)**, demonstrates a dilated left ventricular (LV) cavity (LV end-diastolic diameter [LVEDD] 64 mm), adequately positioned inflow LVAD cannula, continuous aortic regurgitation (AR), and a persistently closed aortic valve.

**Figure 3:**
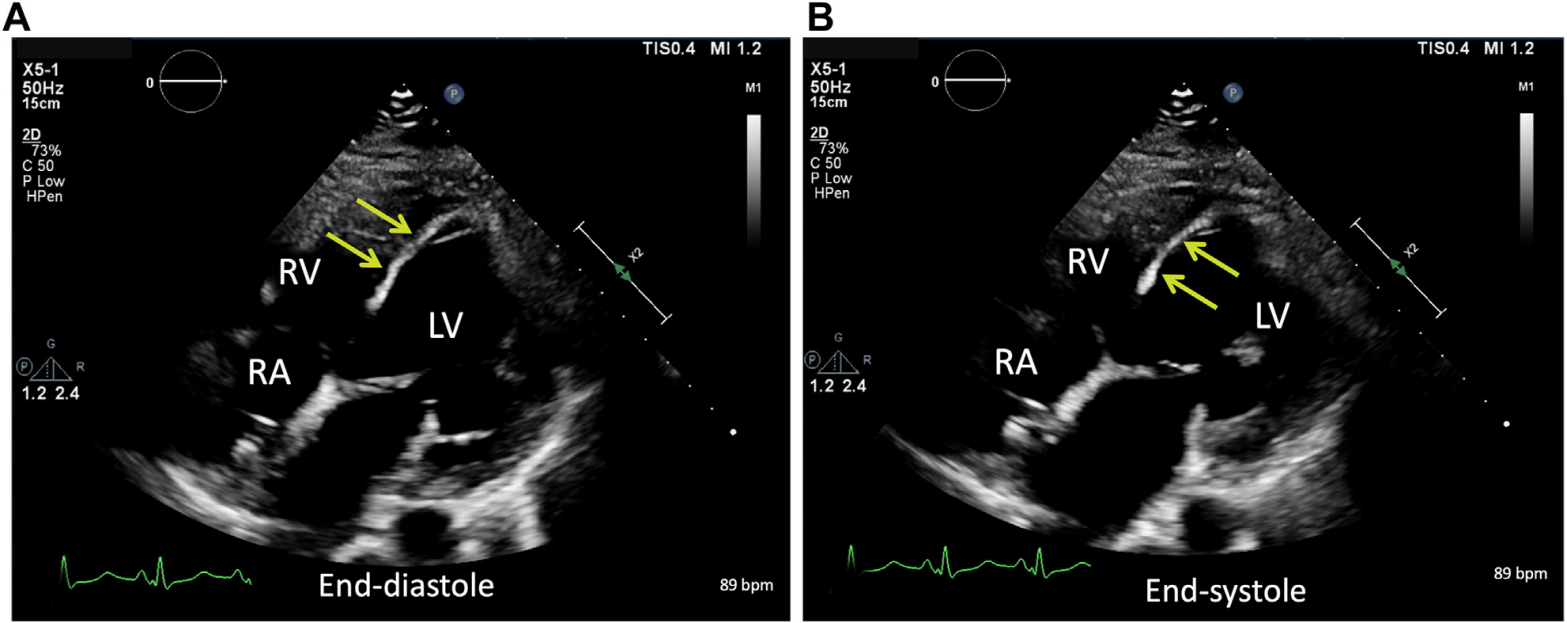
Two-dimensional TTE, right heart–focused apical four-chamber diastolic **(A)** and systolic **(B)** views at admission, demonstrates a relatively small RV compared with the left ventricular size, with the near midline IVS position becoming more rightward at end-systole. *LV*, Left ventricle; *RA*, right atrium.

**Figure 4:**
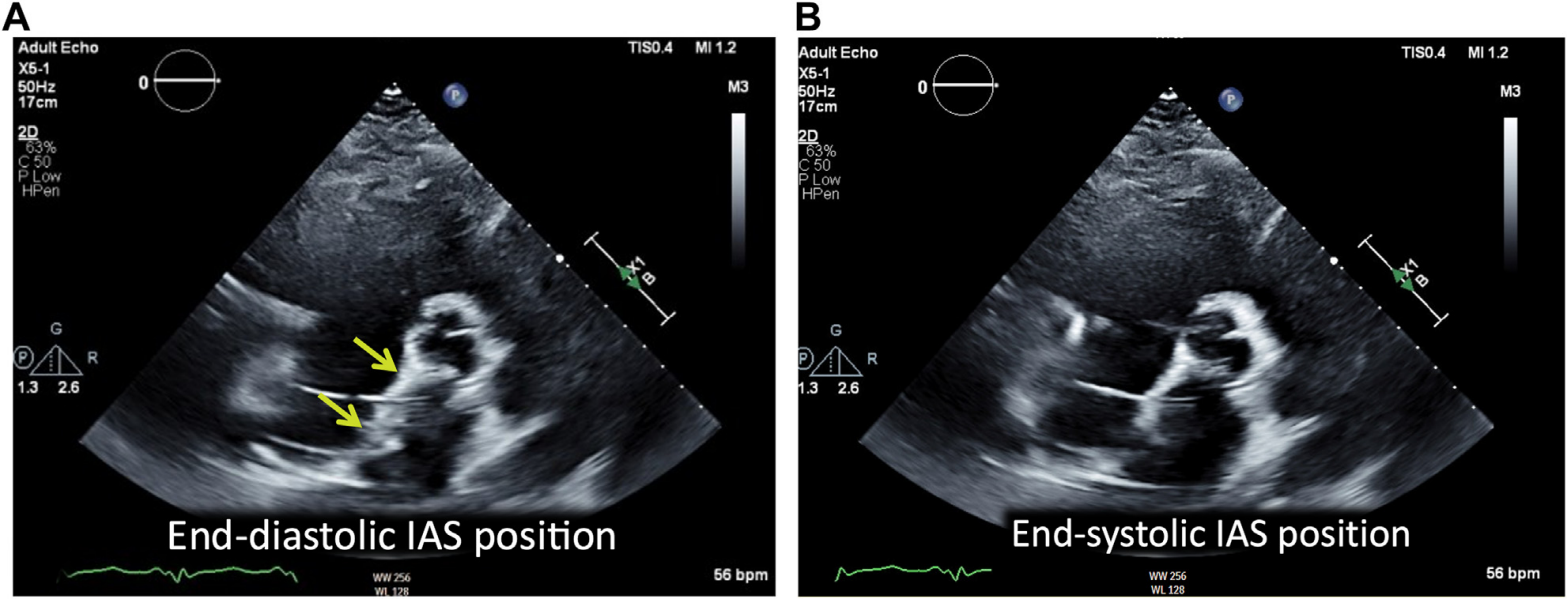
Two-dimensional TTE, basal parasternal short-axis diastolic **(A)** and systolic **(B)** views at admission, demonstrates that the leftward-shifted interatrial septum (IAS) is near midline at end-systole, suggestive of increased right atrial pressure.

**Figure 5:**
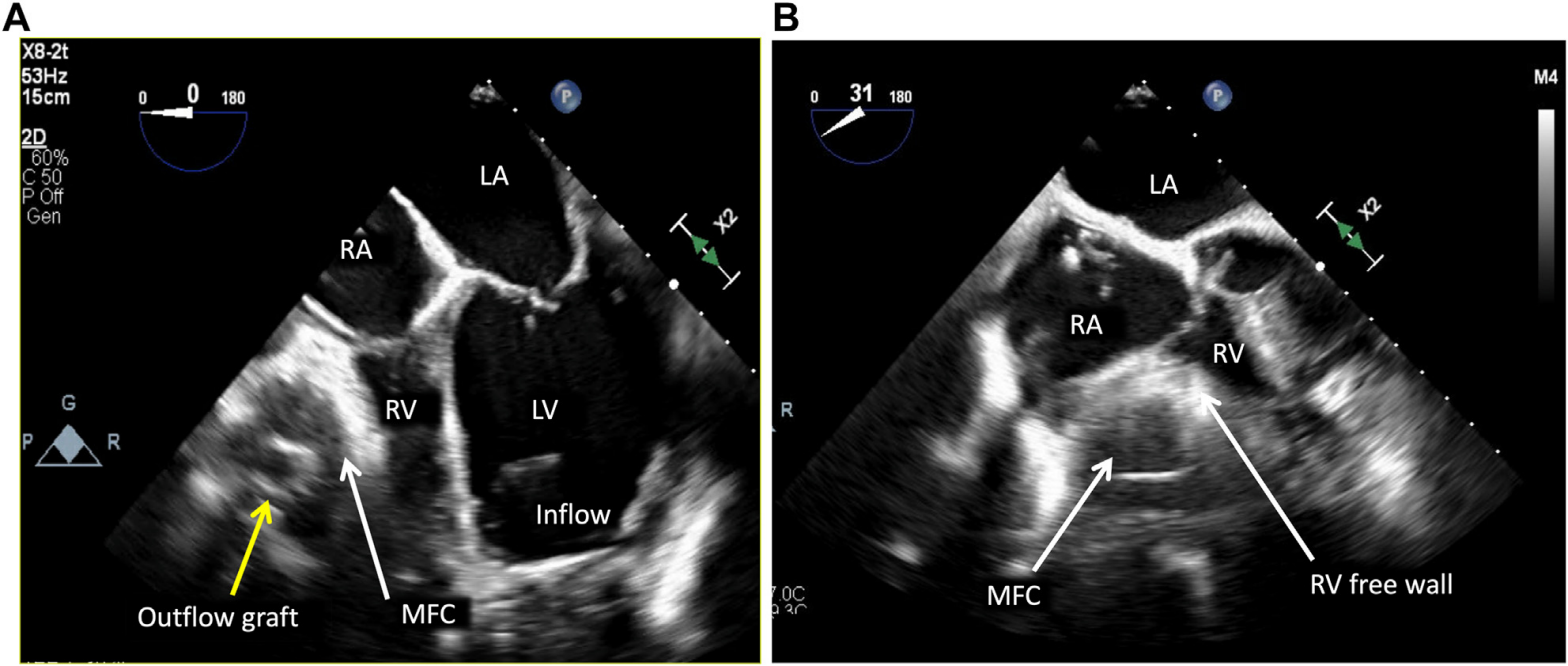
Two-dimensional transesophageal echocardiography, midesophageal long-axis (**A**; 0°) and oblique long-axis (**B**; 31°) views, demonstrates compression of the right ventricular free wall by an MFC *(white arrow)* surrounding the outflow graft *(yellow arrow)* and abutting the right ventricular free wall. *LA*, Left atrium; *LV*, left ventricle; *RA*, right atrium.

Over the ensuing 24 hours, the patient experienced significant alleviation of respiratory symptoms; however, pressure tracings from the pulmonary artery catheter continued to demonstrate persistently elevated right-sided filling pressures with a low cardiac index despite ample diuresis. Shortly thereafter, the patient went on to develop new symptoms of lightheadedness and dizziness, which was surprising given the persistently elevated central venous pressures and adequate diuresis regimen. TTE was performed, which showed a severely dilated left ventricular cavity with left ventricular end-diastolic dimension of 64 mm, severely reduced left ventricular ejection fraction, and a midline-to-rightward interventricular septum (IVS; Figures 2 and 3, Videos 1 and 2). The aortic valve remained closed throughout the cardiac cycle, suggesting inadequate left ventricular unloading by the LVAD (Video 3). Color and continuous-wave Doppler demonstrated no significant tricuspid regurgitation or tricuspid regurgitation gradient. Although subcostal windows were difficult and assessment of the inferior vena cava was not possible, apical views showed a leftward shift of the interatrial septum consistent with increased right atrial pressure (Figure 4, Video 4). Initial TTE also demonstrated a very small right ventricular cavity, which was not expected given the clinical picture of overload and elevated right-sided filling pressures on invasive hemodynamics. There was a moderate-sized fluid collection surrounding the LVAD outflow graft and compressing the right ventricular free wall, better seen on follow-up transesophageal study (Figure 5, Video 5 and 6).

**Figure 6:**
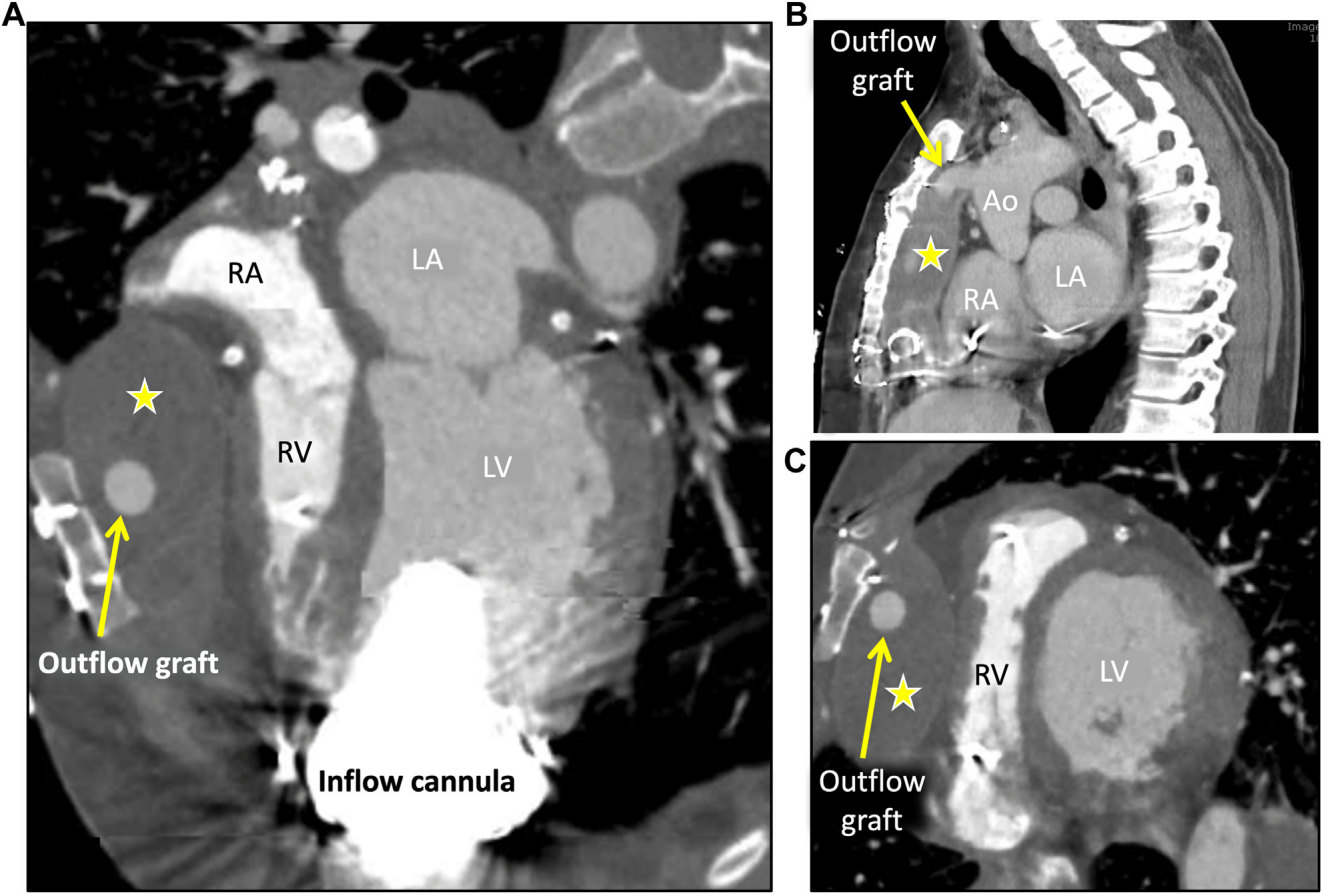
CCT, multiplanar reconstructions in oblique axial **(A)**, modified sagittal, atrial **(B)**, and ventricular **(C)** level display, demonstrates the anteriorly located MFC *(star)* anterior to the RV and right atrium (RA), immediately beneath the sternum, from the level of the anastomosis between the outflow cannula *(arrow)* with the ascending aorta (Ao) and extending inferiorly to the level of the inflow cannula with a mass effect on the right ventricular free wall, tricuspid annulus, and RA.

**Figure 7:**
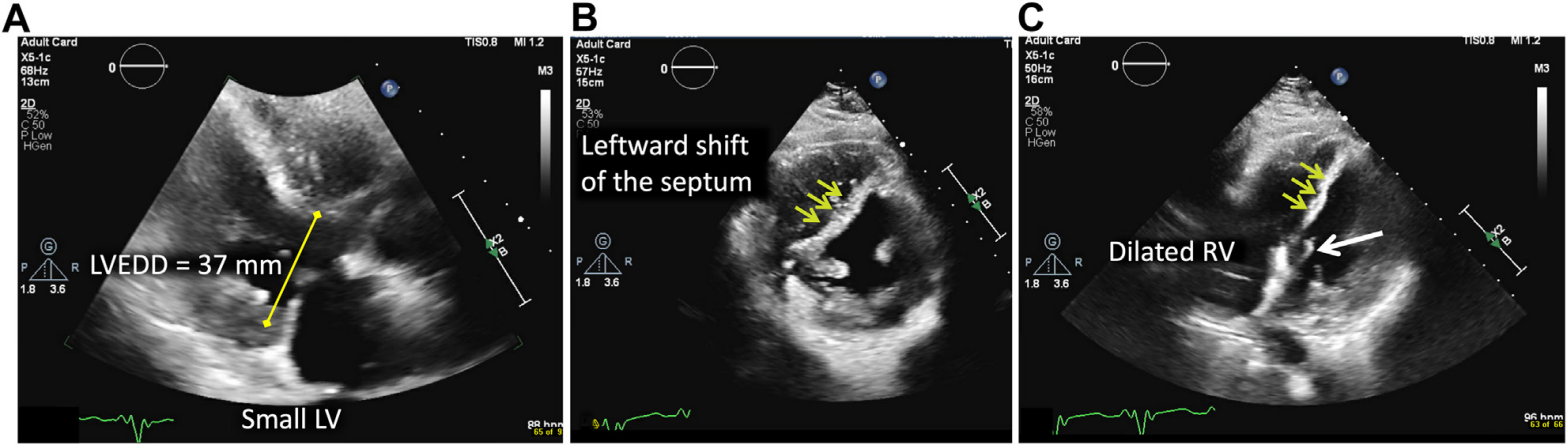
Two-dimensional TTE following percutaneous drainage of the MFC, parasternal long-axis **(A)**, short-axis **(B)**, and apical four-chamber **(C)** diastolic views, demonstrates a smaller left ventricular cavity (left ventricular end-diastolic diameter [LVEED], 37 mm), new leftward position of the IVS *(yellow arrows)*, and new dilation of the RV; also seen is tethering of the mitral valve leaflets *(white arrow)*. *LV*, Left ventricle.

To better evaluate the mediastinal fluid collection (MFC) and assess the patency of the outflow graft, cardiac computed tomography (CCT) was ordered. CCT demonstrated a moderate-sized MFC surrounding the outflow graft and extending from the ascending aorta at the site of outflow cannula anastomosis to the LVAD inflow cannula, anterior to the right ventricle (RV), with evidence of extrinsic right ventricular free wall compression during the cardiac cycle (Figure 6, Videos 7-9). These findings raised concern for tamponade-like physiology, and therefore intravenous diuresis therapy was held. The patient subsequently underwent interventional radiology–guided MFC aspiration for diagnostic and therapeutic purposes, but only 15 mL of clear fluid could be removed. No organisms were seen on gram staining or culture. Repeat TTE shortly after the procedure revealed a leftward shift in the IVS, small left ventricular cavity size, and a dilated RV (Figure 7). Postprocedural TTE also showed significant tethering of the mitral valve leaflets, which suggested exaggerated left ventricular offloading (Videos 10 and 11). Because of these findings on TTE, LVAD speed was accordingly reduced to 2,760 rotations/min, and the patient’s symptoms of lightheadedness and dizziness diminished. Repeat RHC was performed (Table 2), which showed reduced right-sided pressures with decreased wedge pressure and improved cardiac index. These results confirmed the hemodynamic significance of the MFC, which was in fact compressing the RV and preventing filling. The patient was discussed in multidisciplinary heart failure and transplantation rounds, and the decision was made to list them for heart transplantation. Six months later, the patient received a new heart and is currently doing clinically well. Intraoperative samples of the MFC were consistent with a sterile seroma.

**Table 2:** RHC results after 15 mL fluid removal, LVAD speed 2,760 rotations/min

Parameter	Value
Right atrial pressure, mm Hg	5
Right ventricular systolic/diastolic pressure, mm Hg	25/5
PA systolic/diastolic pressure, mm Hg	25/10 (mean, 15)
PCWP, mm Hg	8
PA saturation, %	67
Fick cardiac output, L/min	5.2
Fick cardiac index, L/min/m	2.6
SVR, dyne · s ·cm^−5^	861

*PA*, Pulmonary artery; *PCWP*, pulmonary capillary wedge pressure; *SVR*, systemic vascular resistance.

## Discussion

In 2022, more than 2,500 LVADs were implanted in the United States.^2^ Although many of these devices serve as a bridge to transplantation, a growing number, in recent years, have been implanted as destination therapy. The leading causes of readmission after LVAD implantation include bleeding, infection, heart failure, arrhythmias, pump malfunction, and thrombosis.^3^ Heart failure includes both right heart failure, which can be a late complication in about 11% of patients according to published studies, and left heart failure, which can be due to inadequately treated left heart failure.^4^ The LVAD device operates at a fixed speed, measured in rotations per minute, and cardiac output is dependent on this speed, making LVAD optimization critical to patient quality of life and functional capacity. Optimization of LVAD hemodynamics plays an important role in the treatment of patient symptoms and reduction of readmission rates.^2^^,^^3^ From the TTE perspective, LVAD hemodynamics are optimized when the left ventricular cavity is unloaded, the septum is midline, the aortic valve opens intermittently, and there is minimal mitral regurgitation.

In the setting of increased jugular venous pressure and peripheral edema noted on admission, our patient was initially treated with intravenous diuretics because the diagnosis was presumed to be heart failure and volume overload. Initial TTE showed a dilated left ventricular cavity with a midline-to-rightward septum, suggesting an inadequately unloaded left ventricle even though the aortic valve was closed. The appearance of the left ventricle and septum was similar to that noted on prior transthoracic echocardiography examinations dating back ≥2 years. In addition, Although the inferior vena cava was difficult to see on TTE, the interatrial septum was noted to be leftward, consistent with elevated right atrial pressures This was later confirmed by RHC. Interestingly, the RV on the admission TTE was not dilated, which was discordant with elevated right atrial pressures noted on RHC. Although the patient’s symptoms initially improved with diuretics, the patient quickly developed symptoms of low preload (dizziness and lightheadedness). It is important to note that at this point only data from RHC and TTE were available. From the transthoracic images, the significance of the fluid collection along the right ventricular free wall could not be fully appreciated. It was the larger field of view offered by cross-sectional imaging that demonstrated that the extrinsic compression of the RV by the fluid collection likely caused the patient’s symptoms.

To understand the association between the seroma and heart failure development in our patient, it is important to revisit the mechanisms underlying right ventricular failure in post-LVAD patients. Immediately following LVAD implantation, hemodynamic parameters are radically altered, leading to marked improvements in left-sided filling pressures, decreased right ventricular afterload, and increased systemic circulation. Although these adaptations play a critical role in treatment of cardiogenic shock, they can also lead to a significant increase in right ventricular preload. Initially, the RV attempts to adapt to the increase in preload by increasing its stroke volume. The extent to which the RV can manage this added stress and preserve the hemodynamic balance is dependent on its functional reserve at the time of LVAD implantation.^5^ If the RV is unable to maintain the increase in preload because of increased LVAD speeds and residual hypervolemia, it begins to dilate and fail. If this process remains unmitigated, the resulting increase in venous congestion can lead to renal hypoperfusion, activation of the renin-angiotensin-aldosterone system^6^ triggering a cascade of responses, leading to volume expansion, elevated systemic vascular resistance, and ultimately compromised LVAD efficiency. Another important factor contributing to the development of right ventricular failure is the loss of ventricular interdependence. An intact pericardium and normal septal motion are crucial for maintaining this interdependence. LVAD surgery involves a pericardiotomy, and the procedure itself can alter the function of the IVS, both of which play a central role in the pathophysiology of right ventricular failure.^7^

Our patient initially exhibited no clinical or hemodynamic signs of right ventricular failure; instead, the patient’s presentation was suggestive of obstructive shock physiology. Signs of right ventricular failure manifested only following intervention to address the MFC. Compression of the right ventricular free wall by the MFC caused a tamponade physiology state that prevented the RV from dilating while also maintaining elevated right-sided filling pressures. Similar cases of external compression have been described in pectus excavatum deformities, in which sternal compression on the right ventricular free wall can lead to significant right ventricular dysfunction.^8^^,^^9^ The MFC and the pressure it exerted prevented the RV from dilating, causing it to act like a conduit, which in turn helped preserve the integrity of the LVAD circuit at the set speed in the months before patient presentation. Concurrently, the increased right atrial and central venous pressures resulting from right ventricular compression by the MFC also caused a retrograde increase in volume overload and subsequent elevation in left ventricular afterload. This accounted for the elevated systemic vascular resistance and pulmonary capillary wedge pressure observed on initial RHC, as well as the low-flow alarms detected during the initial LVAD interrogation. Furthermore, TTE demonstrated reduced LVAD efficacy, evidenced by the absence of aortic valve opening and a midline-to-rightward shift of the IVS.

**Figure 8:**
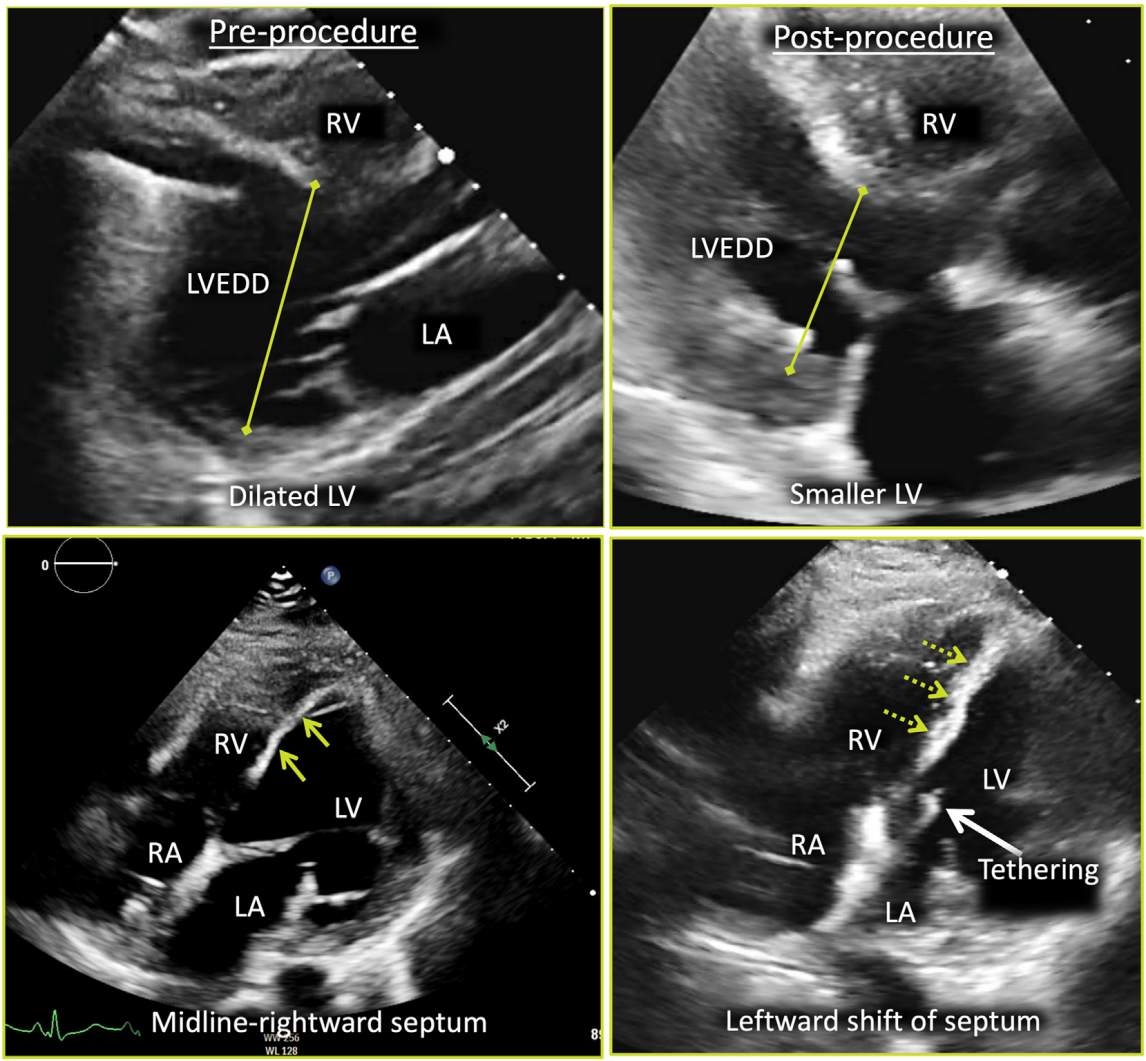
Two-dimensional TTE preprocedure *(left)* and postprocedure after drainage of the MFC *(right)*, parasternal long-axis *(top)* and right heart–focused apical four-chamber *(bottom)* views, demonstrates the reduction in left ventricular cavity, dilation in right ventricular cavity, leftward shift of the IVS *(yellow arrows)*, and tethering of the mitral valve leaflets *(white arrow)*. *LA*, Left atrium; *LV*, left ventricle; *LVEDD*, left ventricular end-diastolic diameter; *RA*, right atrium.

Once the seroma was identified on CCT, percutaneous removal was attempted. Although only 15 mL of fluid was successfully removed from the space, the impact on hemodynamics and imaging findings (Figure 8) was significant. As evidenced by follow-up TTE, the RV was suddenly allowed to expand, rapidly raising right ventricular preload and unmasking significant right ventricular failure. Even though the MFC was not large, we hypothesize that the impact of the fluid removal on an already diseased RV mimicked the phenomenon sometimes observed in cases of pericardial decompression syndrome, a rare complication that occurs after the rapid removal of a large volume of pericardial fluid, resulting in significant right ventricular dilation, failure, and worsening hemodynamics. The exact mechanism of this phenomenon is not fully understood, but several studies have suggested that the sudden increase in venous return, right ventricular expansion, and preload-afterload mismatch immediately following pericardiocentesis are potential contributing factors.^10^ Because the LVAD speed remained constant, pericardiocentesis caused a pericardial decompression syndrome–like state that in turn resulted in a decrease in left ventricular preload, shifting the IVS to the left, decreasing the left ventricular cavity size, and tethering the mitral valve leaflets. It is very likely that this was further exacerbated by aggressive intravenous diuresis therapy in the days leading to pericardiocentesis. This conclusion is supported by the precipitous drop in pulmonary capillary wedge pressure following decompression of the pericardial space and diuretic therapy (Table 2). Hemodynamic equilibrium was restored by decreasing the LVAD speed, as this allows left ventricular filling and preload restoration. Subsequent TTE demonstrated a return to baseline left ventricular dimensions and resolution of mitral valve tethering. At this point, the advanced heart failure team entertained several options for a durable resolution. One approach discussed was surgical explantation of the current LVAD and replacement with a new LVAD. The second approach involved listing the patient for heart transplantation, which the patient underwent a few months later.

Seromas or MFCs within the space between the LVAD outflow and the covering biomaterial graft caused by thrombofibrotic exudate have been previously described.^11^ Some sources suggest that a semiporous outflow graft may permit the escape of proteinaceous material, which, in a subset of patients for reasons yet to be understood, becomes sequestered within the nonporous parts of the outflow graft. This trapped material within the seroma lacks the capacity for reabsorption, further complicating the phenomenon.^11^ Notably, the operative report from the patient’s subsequent transplantation surgery did not document any abnormalities in the appearance of the outflow graft or provide insights into a potential underlying cause. Seromas associated with LVAD outflow grafts have been previously documented as a cause of outflow graft obstruction, potentially leading to pump failure and thromboembolism.^12^ However, the occurrence of seromas leading to cardiac tamponade is exceedingly rare. In one notable example, a late-onset mediastinal seroma developing after thymoma surgery caused cardiac tamponade.^13^ Imaging findings on echocardiography and CCT in this case were similar, and the patient underwent percutaneous drainage of the seroma, leading to resolution of symptoms. Cardiac tamponade in the LVAD population is rare, with most cases occurring immediately postoperatively because of bleeding and hemopericardium formation.^14^ Late tamponade in the LVAD population is therefore extremely rare. Our case represents a unique instance of late-onset tamponade physiology secondary to a graft-associated seroma in an LVAD patient.

## Conclusion

We present a case of an LVAD-associated seroma encasing the outflow graft and resulting in substantial extrinsic compression of the RV. This compression induced cardiac tamponade, which was elucidated through the combined use of echocardiography and cross-sectional imaging. This case underscores the essential role of a systematic, stepwise evaluation in diagnosing patients with LVAD malfunctions and emphasizes the diagnostic value of integrating echocardiographic and cross-sectional imaging modalities in diagnosing this very rare LVAD complication.

## Ethics Statement

The authors declare that the work described has been carried out in accordance with The Code of Ethics of the World Medical Association (Declaration of Helsinki) for experiments involving humans.

## Consent Statement

Complete written informed consent was obtained from the patient (or appropriate parent, guardian, or power of attorney) for the publication of this study and accompanying images.

## Funding Statement

The authors declare that this report did not receive any specific grant from funding agencies in the public, commercial, or not-for-profit sectors.

## Disclosure Statement

The authors report no conflict of interest.

## Supplementary Data

Video 1 Two-dimensional TTE at admission, parasternal long-axis view, demonstrates a dilated left ventricular cavity, adequately positioned inflow LVAD cannula, and severe global left ventricular systolic dysfunction with persistently closed aortic valve.

Video 2 Two-dimensional TTE, apical four-chamber view at admission, demonstrates a relatively small RV compared with the left ventricular size, severe global left ventricular systolic dysfunction, and a near midline IVS diastolic position that becomes more rightward at end-systole.

Video 3 Two-dimensional TTE at admission, parasternal long-axis view with color flow Doppler, demonstrates continuous aortic regurgitation with a persistently closed aortic valve.

Video 4 Two-dimensional TTE, basal parasternal short-axis view at admission, demonstrates that the leftward shifted interatrial septum is near midline at end-systole, suggestive of increased right atrial pressure.

Video 5 Two-dimensional transesophageal echocardiography, midesophageal long-axis (0°) view, demonstrates a dilated left ventricle with severely reduced left ventricular systolic function, a relatively small right ventricular cavity, normally positioned LVAD cannula, pacer wires, and compression of the right ventricular free wall by an MFC surrounding the outflow graft and abutting the right ventricular free wall.

Video 6 Two-dimensional transesophageal echocardiography, midesophageal oblique long-axis (31°) view, demonstrates compression of the right ventricular free wall by an MFC surrounding the outflow graft and abutting the right ventricular free wall.

Video 7 CCT, cine stack of the multiplanar reconstructed axial display, demonstrates the comprehensive extent of the MFC seen to be surrounding and encapsulating the entire LVAD outflow graft, representing a graft-associated seroma and compressing the right heart.

Video 8 CCT, cine stack of the multiplanar reconstructed sagittal display, demonstrates the comprehensive extent of the MFC seen to be surrounding and encapsulating the entire LVAD outflow graft, representing a graft-associated seroma and compressing the right heart.

Video 9 CCT, sagittal display at the level of the midventricular cavity, reconstructed cine loop from each cardiac cycle, demonstrates the large left ventricular/right ventricular ratio, reduced left ventricular systolic function, and compression of the right ventricular free wall from the MFC (LVAD outflow graft with surrounding seroma).

Video 10 Two-dimensional TTE following percutaneous drainage of the MFC, parasternal long-axis view, demonstrates a smaller left ventricular cavity, larger right ventricular cavity, new leftward position of the IVS, and new tethering of the mitral valve leaflets.

Video 11 Two-dimensional TTE following percutaneous drainage of the MFC, right heart–focused apical four-chamber view, demonstrates a smaller left ventricular cavity, larger right ventricular cavity, new leftward position of the IVS, and new tethering of the mitral valve leaflets.
